# A Diagnostic Classifier Based on Circulating miRNA Pairs for COPD Using a Machine Learning Approach

**DOI:** 10.3390/diagnostics13081440

**Published:** 2023-04-17

**Authors:** Shurui Xuan, Jiayue Zhang, Qinxing Guo, Liang Zhao, Xin Yao

**Affiliations:** 1 Department of Respiratory & Critical Care Medicine, The First Affiliated Hospital of Nanjing Medical University, 300 Guangzhou Road, Nanjing 210029, China; 2 Department of Neurosurgery, The First Affiliated Hospital of Nanjing Medical University, 300 Guangzhou Road, Nanjing 210029, China; 3 Department of Neurosurgery, The Affiliated Brain Hospital of Nanjing Medical University, 264 Guangzhou Road, Nanjing 210029, China

**Keywords:** COPD, miRNA, machine learning, diagnostic model

## Abstract

Chronic obstructive pulmonary disease (COPD) is highly underdiagnosed, and early detection is urgent to prevent advanced progression. Circulating microRNAs (miRNAs) have been diagnostic candidates for multiple diseases. However, their diagnostic value has not yet been fully established in COPD. The purpose of this study was to develop an effective model for the diagnosis of COPD based on circulating miRNAs. We included circulating miRNA expression profiles of two independent cohorts consisting of 63 COPD and 110 normal samples, and then we constructed a miRNA pair-based matrix. Diagnostic models were developed using several machine learning algorithms. The predictive performance of the optimal model was validated in our external cohort. In this study, the diagnostic values of miRNAs based on the expression levels were unsatisfactory. We identified five key miRNA pairs and further developed seven machine learning models. The classifier based on LightGBM was selected as the final model with the area under the curve (AUC) values of 0.883 and 0.794 in test and validation datasets, respectively. We also built a web tool to assist diagnosis for clinicians. Enriched signaling pathways indicated the potential biological functions of the model. Collectively, we developed a robust machine learning model based on circulating miRNAs for COPD screening.

## 1. Introduction

Currently, chronic obstructive pulmonary disease (COPD) is one of the top three causes of death worldwide, and 90% of these deaths occur in low- and middle-income countries [1]. COPD is characterized by persistent respiratory symptoms, almost irreversible obstruction of airflow, and aggressive disease progression [2]. The Global Initiative for Chronic Obstructive Lung Disease (GOLD) recommended that the post-bronchodilator forced expiratory volume in 1s (FEV1) to forced vital capacity (FVC) ratio < 0.7 is mandatory to establish the diagnosis of COPD [2]. Considering the fixed ratio of forced spirometry may lead to over-diagnosis in the elderly and underdiagnosis in adults [3], effective molecular biomarkers could be prior in early detection and timely identification of COPD. The molecular mechanisms of COPD are complex; for example, mitochondria autophagy initiated programmed necrosis and cellular senescence [4], epigenetic dysregulation of DNA methylation state [5], and microRNA (miRNA) dysregulation [6].

miRNAs are small non-coding RNA molecules found in tissues and body fluids. Expression profiling of lung tissue from COPD patients and smokers without COPD has revealed several differentially expressed miRNAs [7]. Circulating miRNA biomarkers can be collected without the need for invasive tissue biopsy, and their bioactivity usually stays stable in varied conditions, e.g., repetitive freezing and thawing cycles [8]. Furthermore, miRNAs exhibit superiority in simple chemical structures without post-processing modifications [9], which can be applied as ideal biomarkers and predictive molecules. However, most studies were based on the expression levels of miRNAs which may vary significantly when transferred to other quantification machines. This makes determining an exact cutoff value for diagnosis difficult in practical application. Therefore, the development of novel robust and cross-platform diagnostic biomarkers is urgently needed.

Machine learning is a subfield of artificial intelligence which refers broadly to constructing predictive models and identifying informative groupings to recognize the data using computation [10]. Machine learning has progressively enhanced the capability to search biological signatures from massive and high-dimensional biological sample data to improve clinical diagnosis and therapeutic strategies [11]. To date, the application of machine learning models has been involved in the diagnosis and prognosis estimation of axial spondyloarthritis [12], breast cancer [13], malaria [14], primary immunodeficiency disease [15], etc.

In this study, we constructed miRNA pairs according to the relative expression differences of circulating miRNAs and built machine learning models for COPD diagnosis. We assessed the predictive performance of these models and identified an optimal classifier as a diagnostic tool.

## 2. Materials and Methods

### 2.1. Data Collection and Pre-Processing

Two publicly available datasets profiling miRNA expressions from human pe-ripheral blood samples used in the present study, GSE61741 and GSE70080, were downloaded from the GEO database. We systematically searched two publicly available databases, NCBI GEO and ArrayExpress, using all possible combinations of the following search terms: (a) “COPD” OR “chronic obstructive pulmonary disease”; (b) “microRNA” OR “miRNA”. Then, we manually screened the datasets with samples of human peripheral blood. Finally, the GSE61741 and GSE70080 datasets were selected. The GSE61741 cohort included 94 healthy donors and 47 COPD patients [16]. This dataset was based on the febit Homo Sapiens miRBase 13.0 platform. GSE70080 cohort detected the miRNA expressions from 16 healthy donors and 16 COPD patients using the TaqMan Low Density Arrays Cards platform, which is based on real-time PCR reactions [17]. Raw data from the two datasets were downloaded, and the probe IDs of the expression matrices were re-annotated to match the latest miRBase (v22) names using the miRBaseConverter R package [18]. The intersecting miRNAs between thr GSE61741 and GSE70080 datasets were further retained. According to the original publication, missing values in the expression profiles of GSE70080 cohort represented the low-expressed RNA copies and were replaced with the minimum value of the matrix in this study. Moreover, lower expression abundance miRNAs with a proportion of minimum value over 70% across samples from healthy donors or COPD patients were excluded from the next analysis.

### 2.2. Construction of miRNA Pair-Based Signature

The expression levels of two miRNAs in a specific blood sample were compared pairwise to generate a score, 0 or 1. A miRNA pair score of 0 was assigned when the expression of miRNA-A was less than miRNA-B. Otherwise, the miRNA pair score was 1. The original expression profiles of the datasets were then transformed into miRNA pairs-samples matrices consisting of only 0 and 1. Then GSE61741 and GSE70080 cohorts were comparable and merged into a whole dataset based on the intersecting miRNA pairs.

### 2.3. Machine Learning Model Development and Evaluation

To strike a balance between the applicability and robustness of the miRNA pair-based model, we first performed feature selection on the matrix described above. The ability of each miRNA pair to distinguish between COPD patients and healthy donors was measured using the area under the curve (AUC); miRNA pairs with AUC > 0.7 were obtained.

Other three feature selection algorithms, including information gain, maximum relevancy minimum redundancy (MRMR), and Boruta, were used before building our machine learning models. Briefly, information gain can pick the most informative and significant features based on the entropy values of features [19]. MRMR is a supervised feature selection model that identifies factors maximum relevant to the target classes along with maximally mutual to other features [20]. Boruta utilizes a statistical significance test to eliminate features with less importance values than that of shadow features [21]. The top 20 variables were selected from both information gain and maximum relevancy minimum redundancy methods. miRNA pairs that met the Boruta screening criteria were also selected. After that, the intersected miRNA pairs from the four feature selection methods were finally identified as key variables and further used as input for modeling.

Before building the machine learning models, the whole cohort was divided into training and testing sets. The random sampling conducted the 70/30 split stratified with the given diagnosis information. We also used 10-fold cross-validation, a well-established resampling method, to better understand the performance of a model. Then, we performed seven machine learning algorithms, including K-nearest neighbors (k-NN), support vector machine (SVM), random forest, Naive Bayes, decision tree, eXtreme Gradient Boosting (XGBoost), and Light Gradient-Boosting Machine (LightGBM), to construct prediction models based on the selected features. The parameters of each model were tuned with a grid search approach according to their official documentation.

### 2.4. Study Subjects

Ethics approval was obtained from The First Affiliated Hospital of Nanjing Medical University (FAHNMU) (2019-SR-371). Subjects were categorized as healthy controls (*n* = 20) and COPD patients (*n* = 25). COPD was defined according to the GOLD criteria (FEV1/FVC < 70%). Patients were excluded if they presented with other diseases, including severe cardiovascular disease, uncontrolled high blood pressure, bleeding, hepatic failure, renal failure, rheumatoid immune disease, and malignant tumors.

### 2.5. Quantitative Real-Time Polymerase Chain Reaction (qRT-PCR)

Total RNA was extracted from peripheral blood samples using TRIzol^TM^ LS reagent (Invitrogen, Waltham, MA, USA). The primers (one RT primer and a pair of qPCR primers for each set) specific for miRNAs were designed by Guangzhou RiboBio Co., Ltd. (Guangzhou, China). The primer sequences were patented. miRNA amplification was conducted using Bulge-Loop™ miRNA qRT-PCR Starter Kit (cat. no. R11067.3; Guangzhou RiboBio, Guangzhou, China) according to the manufacturer’s instructions. In brief, miRNA was firstly reverse transcribed to complementary DNA (cDNA) in the condition of 42 °C for 60 min, followed by 70 °C for 10 min. qRT-PCR was carried out in triplicate in 384-well plates by QuantStudio5 real-time PCR system (Applied Biosystems, Waltham, MA, USA). The following thermocycling conditions were used: initial denaturation at 95 °C for 10 min, followed by 40 cycles of 95 °C for 2 s, 60 °C for 20 s, and 70 °C for 10 s. Ct values were obtained to represent the expressions of miRNAs in each sample.

### 2.6. Statistical Analyses

Analyses and figure generation were conducted using the R software (v4.2.0, The R foundation, Vienna, Austria; www.r-project.org, accessed on 1 February 2023). Differentially expressed miRNAs in different groups were identified using the limma R package [22]. Machine learning model construction and validation were performed using tidymodels ecosystem R packages. Pathway and biological process enrichment analysis was carried out using Metascape [23]. The ontology term with *p*-value < 0.01 and gene count > 3 was selected as an enriched pathway. AUC, sensitivity, specificity, positive predictive value (PPV), negative predicted value (NPV), and overall accuracy were calculated to evaluate models.

## 3. Results

### 3.1. Dataset Description

**Figure 1:**
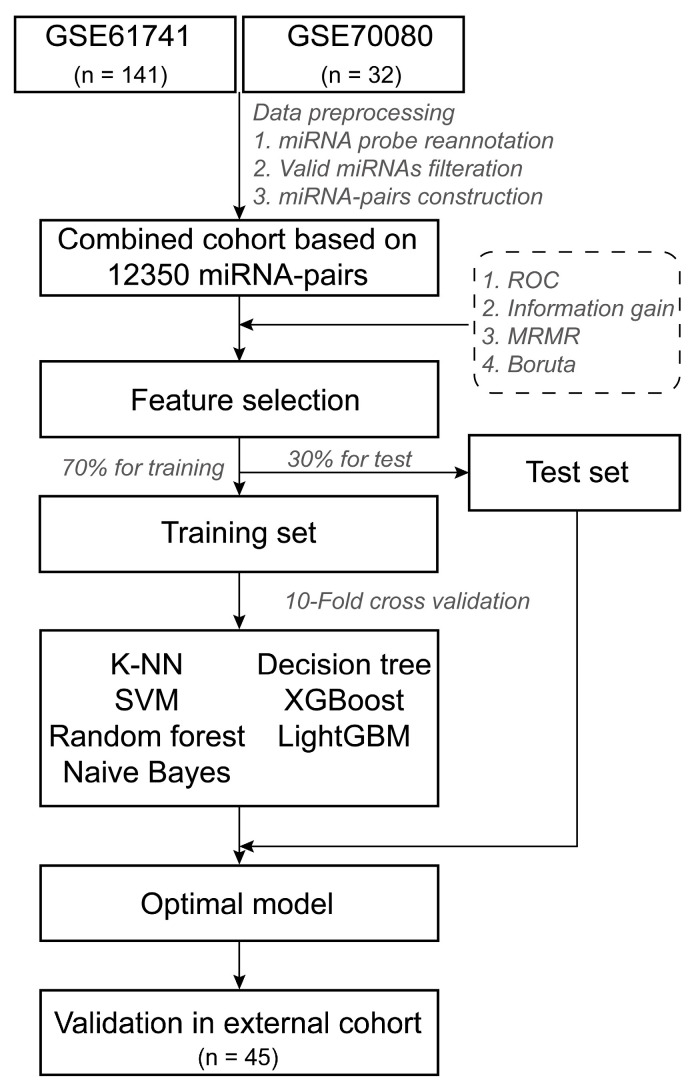
An overall flow chart of the study.

To construct miRNA pair-based expression matrix of COPD patients and healthy controls, we incorporated two independent miRNA datasets based on microarray and qRT-PCR platforms. The workflow of the study is shown in Figure 1. A total of 63 COPD and 110 normal blood samples were obtained from GSE61741 and GSE70080 cohorts. The quality control process yielded 840 and 253 valid miRNA probes in GSE61741 and GSE70080 datasets, respectively. These two cohorts shared 233 intersecting miRNA probes. We further removed miR-196b-5p and miR-204-5p from the GSE61741 cohort since the proportions of the lowest expression values were over 70%. This step resulted in 231 qualified miRNAs obtained from GSE61741 dataset. Similarly, 175 miRNA probes were retained in the GSE70080 dataset. Finally, a total of 173 miRNAs were overlapped in both GSE61741 and GSE70080 cohorts.

### 3.2. The Expression of Single miRNAs and miRNA Signature Failed in COPD Diagnosis

**Figure 2:**
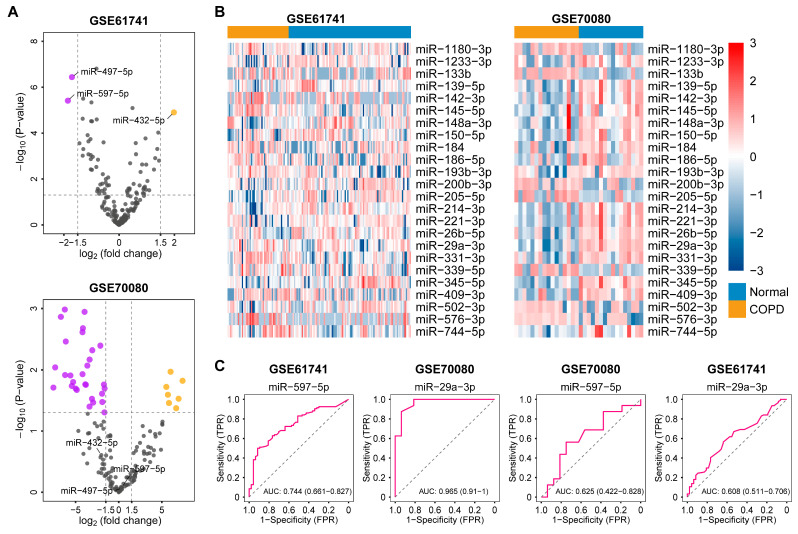
Comparison of the miRNA expression patterns and evaluation of the diagnostic value of miRNAs in two discovery datasets. (**A**) Volcano plots of DEmiRs (|log2FC| > 1.5 and *p*-value < 0.05) in samples from COPD and healthy controls in the GSE61741 and GSE70080 datasets. The yellow dots represent significantly up-regulated miRNAs in COPD, while the purple dots correspond to down-regulated miRNAs; (**B**) Heatmaps of the expression patterns of DEmiRs with a less stringent range (*p*-value < 0.05) between COPD and healthy controls in the two cohorts. Normalized expression values were scaled to the range of −3 to 3; (**C**) Receiver operating characteristic (ROC) curves measuring the performance of two representative miRNAs for predicting COPD and normal controls in the two datasets.

We first explored whether the expression of miRNAs can distinguish COPD patients from normal healthy donors. We identified 62 differentially expressed miRNAs (DEmiRs) (|logFC| > 1.5, *p*-value < 0.05) in the GSE70080 dataset, while only 3 DEmiRs were confirmed in the GSE61741 cohort (Figure 2A) (Table S1). A total of 19 miRNAs were significantly upregulated in COPD patients from GSE70080 dataset, and 43 miRNAs were downregulated in COPD compared with healthy samples. In the GSE61741 dataset, the expression levels of miR-432-5p in COPD were significantly higher than in the healthy group, while miR-497-5p and miR-597-5p were downregulated in the COPD group. We noticed that these three DEmiRs in the GSE61741 dataset were not included in the DEmiRs identified from the GSE70080 cohort.

After setting the criteria to a less stringent range (*p*-value < 0.05), 70 and 62 DEmiRs were calculated in the GSE61741 and GSE70080 datasets, respectively (Table S2). Among them, 24 miRNAs were consistently differentially expressed in the two independent cohorts. However, the expression patterns of these intersecting DEmiRs in different groups were obviously distinct in the two datasets (Figure 2B). The AUC value of miR-597-5p was 0.744 (95% confidence interval (CI): 0.661–0.827), which was the biggest value among all DEmiRs in GSE61741 dataset (Figure 2C). However, the AUC value of miR-597-5p was 0.625 (95% CI: 0.422–0.828) in the GSE70080 dataset. Similarly, the AUC value of miR29a-3p was 0.965 (95% CI: 0.91–1) and 0.608 (95% CI: 0.511–0.706) in the GSE70080 and GSE61741 datasets, respectively. These results indicated that the expression of single miRNAs or miRNA combinations showed unsatisfactory performance in COPD diagnosis among cross-platform cohorts.

### 3.3. Construction of miRNA Pairs and Feature Selection

**Figure 3:**
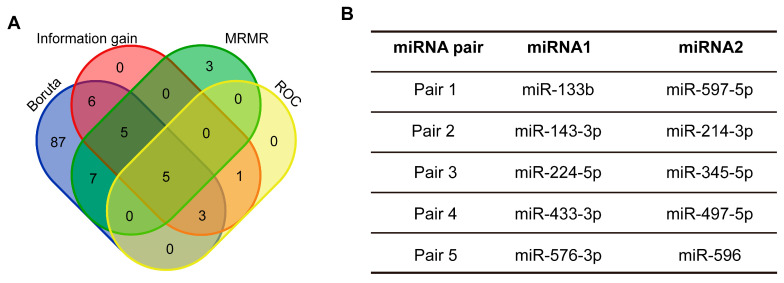
Identification of five miRNA pairs for model development using feature selection methods. (**A**) Venn diagram of overlapping miRNA pairs among four sets filtered by ROC, information gain, maximum relevancy minimum redundancy (MRMR), and Boruta algorithms. The numerical values depicted in the diagram represent the overlapping miRNA quantities among different algorithms; (**B**) Detailed information of the five miRNA pairs.

Next, we focused on developing an effective diagnostic model based on miRNA pairs. The matrix based on 12,350 miRNA pairs was constructed using the 173 overlapped miRNA probes in the two cohorts. The values of miRNA pairs were comparable in each cohort; thus, these two datasets were combined into a whole cohort. Four feature selection methods were applied to determine the miRNA pairs most relevant to the diagnosis, including ROC, information gain, MRMR, and Boruta (Table S3). The ROC selection method yielded nine miRNA pairs with AUC > 0.7. The top 20 miRNA pairs obtained from information gain and MRMR processes were further extracted, respectively. Meanwhile, 113 valid miRNA pairs were identified using the Boruta algorithm. Finally, five miRNA pairs consisting of 10 different miRNAs overlapped among these four selection methods (Figure 3A). Interestingly, some miRNAs among these five pairs were DEmiRs identified above, such as miR-497-5p and miR-597-5p (Figure 3B).

### 3.4. Machine Learning Model Establishment and Evaluation

**Figure 4:**
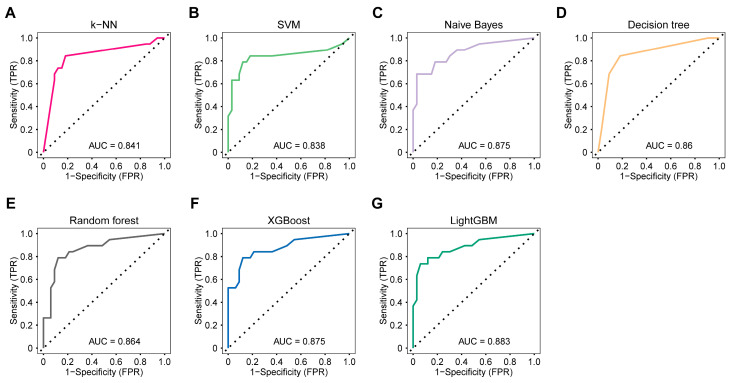
Comparison of the diagnostic performance of seven miRNA pair-based machine learning models. ROC curves measuring the performance of the models for predicting COPD patients and normal controls using (**A**) k-NN, (**B**) SVM, (**C**) Naive Bayes, (**D**) decision tree, (**E**) random forest, (**F**) XGBoost, and (**G**) LightGBM algorithms.

Seven machine learning algorithms were used for the development of diagnostic tools for COPD based on the five miRNA pairs. The whole cohort was divided into training data (*n* = 121) and test data (*n* = 52). The performances of the machine learning methods are shown in Table S4. Among all these models, LightGBM outperformed all the other models with the highest AUC value (0.883, 95% CI: 0.779–0.987), while SVM had the lowest AUC (0.838, 95% CI: 0.697–0.979) (Figure 4A–G). In the test data, 15 of 19 COPD patients (0.789 sensitivity) and 29 of 33 healthy donors (0.879 specificity) were correctly classified using the LightGBM model with an overall accuracy of 0.846. Therefore, the diagnostic signature based on the LightGBM method was selected as the optimal model in the present study.

### 3.5. Validation of the miRNA Pair-Based Model in an External Cohort

**Figure 5:**
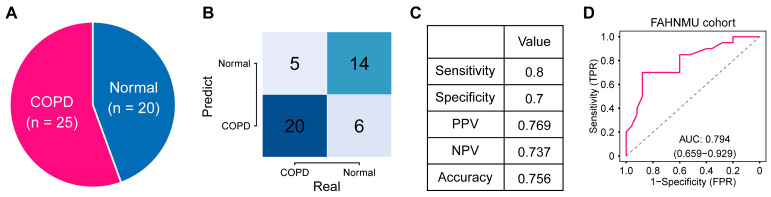
Independent validation of the miRNA pair-based diagnostic model in an external cohort. (**A**) A summary plot of the samples used in the FAHNMU cohort; (**B**) Confusion matrix comparing proportions of predicted COPD and healthy controls in different groups stratified by the miRNA pair-based model; (**C**) Performance of the diagnostic model on the external dataset. PPV, positive predictive value; NPV, negative predictive value; (**D**) ROC curves measuring the predictive performance of the model in the FAHNMU cohort.

We further tested the performance of the miRNA pair-based LightGBM model in the FAHNMU cohort to verify the robustness of the model. The expressions of 10 miRNAs in the model were quantified with Ct values using qRT-PCR. We found that our model correctly classified 20 of 25 COPD patients and 14 of 20 healthy donors (Figure 5A–C) (Table S5). As shown in Figure 5D, the AUC value of the model in the FAHNMU cohort was 0.794 (95% CI: 0.659–0.929). These findings suggested that the predictive performance of the miRNA pair-based LightGBM model was robust and compatible across different detection platforms.

### 3.6. Development of a Web Application for COPD Prediction

We have built a user-friendly web tool to be utilized by clinicians to predict the diagnosis using the LightGBM classifier (https://cav031-liang.shinyapps.io/COPD_ML/, accessed on 1 February 2023). Expression values from microarray or RNA-seq profiles and Ct values from qRT-PCR experiments can be used as input data for the web tool. Select 0 in the sidebar if the expression level of miRNA-A is less than miRNA-B in the “miRNA-A|miRNA-B” pair; otherwise, select 1. The predicted diagnosis and the corresponding estimated probability of a single patient can be easily acquired (Figure S1).

### 3.7. Functional Annotation of the miRNA Pairs in the Model

**Figure 6:**
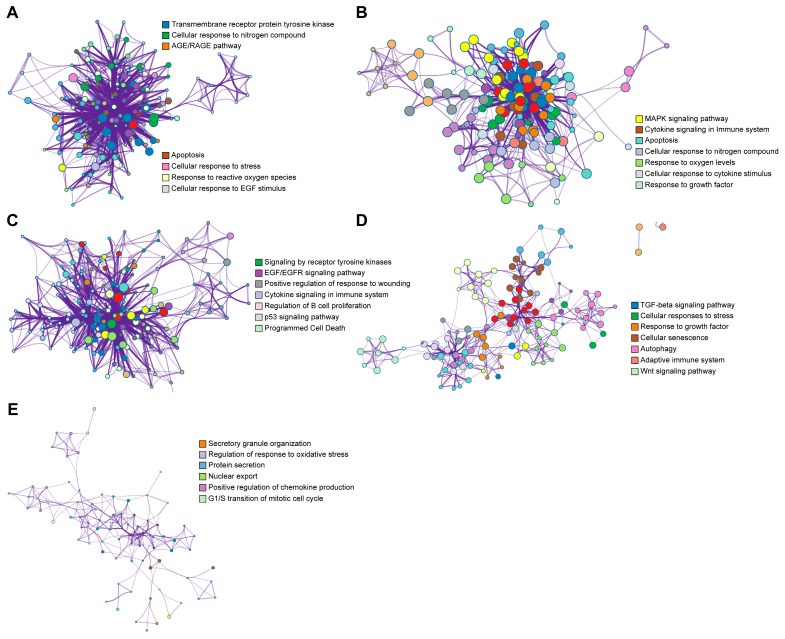
Functional annotations of the five miRNA pairs in the model. (**A**–**E**) Network of signaling pathways and biological processes enriched in the target genes of miRNA pair 1–5. Similar enriched terms are close to each other and connected by edges.

Next, we aimed to explore the biological functions associated with the miRNA pairs in the diagnostic model. Experimentally validated target genes of the five miRNA pairs were obtained from the miRTarBase database [24]. Gene enrichment analysis for each miRNA pair was performed using the Metascape web tool (Table S6). The result showed that the target genes of miRNA pair 1 (miR-133b and miR-597-5p) tended to be strongly associated with cellular response to stress, reactive oxygen, epidermal growth factor, and apoptosis pathways (Figure 6A). Gene terms involved in immune cytokine signaling, apoptosis, oxygen levels, and MAPK signaling pathways were enriched in the miRNA pair 2 (miR-143-3p and miR-214-3p) (Figure 6B). Target genes of miRNA pair 3 (miR-224-5p and miR-345-5p) mainly regulated the EGF/EGFR signaling, wounding response regulation, immune cytokine, and programmed cell death (Figure 6C). Apart from cellular stress and immune cytokine, biological processes, including TGF-beta signaling, cell senescence, and autophagy, were also enriched in the targets of miRNA pair 4 (miR-433-3p and miR-497-5p) (Figure 6D). BH3-only proteins activation and oxidative stress response were significantly associated with miRNA pair 5 (miR-576-3p and miR-596) (Figure 6E).

## 4. Discussion

Along with economic development, the prevalence of COPD is expected to increase with the global population aging [25]. In spite of its prevalence, COPD is underdiagnosed, so quite a few patients do not receive a diagnosis until clinically advanced procession. Hence, early identification and intervention before severe irreversible progression could minimize disability [26]. The potential of circulating miRNAs to be clinical diagnostic biomarkers was raised by their disease-specific expression, rapid detection, and minimal invasiveness. The molecular types commonly utilized as circulating biomarkers are protein, mRNA, and miRNA. Compared with the previous two, miRNA exhibits considerable stability in both structure and function, without undergoing transcriptional modification of mRNA or post-translational modification of protein [27]. In addition, compared to protein detection techniques with higher costs and longer detection time, such as mass spectrometry analysis and enzyme-linked immunosorbent assay (ELISA), methods for miRNA detection are relatively inexpensive and allow for large-scale screening [28,29]. Up to date, miRNAs have emerged as a potential disease modifier in various respiratory diseases, e.g., idiopathic pulmonary fibrosis (IPF) [30] and acute lung injury (ALI)/acute respiratory distress syndrome (ARDS) [31]. Several differentially expressed miRNAs have been identified implicated in potential pathogenic mechanisms of COPD. Hassan et al. revealed the regulatory role of miR-199a-5p in the unfolded protein response (UPR) in the endoplasmic reticulum (ER) stress [32]. The most prevalent miRNA in COPD, the increased miR-223 down-regulated expression levels of the epigenetic modifier histone deacetylase 2 (HDAC2) [33]. Christenson et al. demonstrated that miRNAs altered with regional emphysema severity and modulated pathogenic procedures, e.g., miR-638, might be involved in oxidative stress response and aging pathways in the emphysematous lung [34]. Overall, miRNAs could be a promising component in future COPD screening programs of preventive treatment.

Studies have shown that the expression of single miRNAs or miRNA-based signatures from the peripheral blood samples can function as diagnostic biomarkers for multiple diseases. Latorre et al. developed a novel combination consisting of five miRNAs to diagnose tuberculosis with 91.21% sensitivity and 87.95% specificity [35]. Serum miR-214 expressions were proven to ideally differentiate between neoplastic tumors and healthy controls with an AUC of 0.883 [36]. Leidinger et al. successfully developed a blood-based 12-miRNA signature for the diagnosis of Alzheimer’s disease with an accuracy of 93% [37]. However, the reproducibility of the diagnostic models based on the expression levels of miRNAs in other independent cohorts still remains debatable. For example, circulating miR-21 was proven to be significantly overexpressed in patients with type 2 diabetes, while the up-regulation was not observed in another study [38,39]. Sapre et al. found that the eight miRNAs in blood samples measured by another platform only showed moderate predictive performance in high-risk prostate cancer prediction with an AUC value of 0.62 [40]. The diverging results of the expression-based patterns may be attributed to the different high-throughput technologies and batch effects, including experimental conditions and individual errors [41]. Considering the different ranges of the absolute expression values measured using qRT-PCR, microarray, and RNA-seq platforms, prediction for sample types in other independent datasets using the original parameter will not be applicable. Therefore, the cuff-off of miRNA expression used for specific disease type identification in the clinical application should be determined based on the new large population cohort [42]. Furthermore, high cost and specialized bioinformaticians recruitment hinder the widespread use of high-throughput sequencing platforms in practical testing [43]. Given that both microarray and RNA-Seq can achieve higher resolution of detected miRNA expressions than low-throughput screening, the differentially expressed miRNAs identified using high-throughput sequencing methods in the experimental dataset may be unavailable in the validation cohort. Collectively, the intrinsic limitations of the diagnostic factors based on miRNA quantification suggest that a novel model needs to be developed.

In our study, we collected two independent miRNA expression datasets based on microarray and qRT-PCR platforms, respectively. We noticed the distinct expression patterns of miRNAs between the two cohorts where no significant DEmiR was overlapped. Moreover, the disparate performance of miRNA expression levels in predicting COPD diagnosis in the two datasets suggested the unsatisfied model generalization capability. We provided a novel method based on the comparison of two miRNAs in miRNA pairs instead of the absolute expression values to build a robust predictive model. This approach has remarkable advantages that cannot be overlooked compared with the traditional method. The raw expression profile can be converted to a binary matrix with 0 and 1 without the need for normalization, which ensures reproducibility across different platforms and batch effects. In clinical translational application, the expression difference of miRNA1 and miRNA2 in a single pair can be easily obtained to realize individualized diagnosis for each patient. The annotation and naming schemes of miRNAs have changed with miRBase database iterations [44]. Mature miRNAs naming in the old version changed from “miR/miR ∗” to “-3p/-5p” as suffixes in the recent miRBase versions. Therefore, we re-annotated the miRNA expression profiles of the two datasets used in the present study to obtain the common miRNAs and make the diagnostic model feasible for practical application. It is worth noting that the miRNA pair-based model was trained and tested primarily in publicly available retrospective COPD cohorts. Thus, the model was further validated in our Chinese cohort and still demonstrated effectiveness in distinguishing COPD from healthy donors.

Previous studies have partially verified the expression patterns of the miRNAs in the model we developed for COPD. In a study on the medicine efficacy in the treatment of COPD, a Chinese patent drug was reported to relieve clinical symptoms with higher levels of miR-133b expression in peripheral blood [45]. In COPD-like lung injury caused by sulfur mustard, miR-143-3p was significantly up-regulated and acted as a suitable diagnostic biomarker [46]. miR-497-5p was identified downregulated in COPD plasma [47], while it exhibited a significant increase in exosomes extracted from plasma [48]. The fibroblasts of COPD patients showed significant induction of miR-143-3p after relatively high concentration of TGF-β1 stimulation [49]. COPD is caused by exposure to inhaled particulate matter, including cigarette smoke and air pollutants. Certain exposures differentially regulated miRNA expression *in vivo*. For example, the miR-214-3p–IKK-β axis was involved in cigarette smoke-induced pulmonary inflammation [50]. Additionally, miR-224-5p in plasma exosomes has been clarified related to smoking, especially E-cigarette smoking [51]. Furthermore, air pollution exposure was demonstrated to decrease circulating miR-433-3p levels as well [52].

The biological functions of miRNA target genes implicated in COPD pathogenesis are heterogeneous. Inhaled exposures caused stress leads to lung injury and cellular response to reactive oxygen species [53], which was found significantly enriched in all the five miRNA pairs in our study. Oxidative damage to endothelial and epithelial cells contributes to enhanced cellular senescence [54] and programmed cell death, including autophagy [55] and apoptosis [56]. These disorders were found strongly associated with the miRNA pair 1, 2, 3 and 4 in the model. Additionally, the cellular apoptosis pathway P53 signaling associated with miRNA pair 3 and the “G1/S transition of the cell cycle” pathway enriched in the targets of miRNA pair 5 both play a critical role in regulating cell cycle and maintaining cellular stability [57,58]. Damaged cells may lead to auto-compensatory repair [59,60], which is “positive regulation of response to wounding” mentioned in biological functions associated with miRNA pair 3. Once the degree of damage exceeded the compensatory capacity, progressive tissue destruction and remodeled small-airway walls contribute to persistent airflow obstruction [61]. Immunological response, including the integrated system of innate and adaptive immunity, plays a central role in the development of COPD [62]. All of the constructed five miRNA pairs participated in the inflammatory process. For example, they were implicated in cellular response to cytokine stimulus, i.e., epidermal growth factor (EGF) stimulus signaling was enriched in the target genes of miRNA pair 1 and 3, while miRNA pair 4 corresponds to cytokine stimulation including growth factors and TGF-β. The certain involved inflammatory processes, such as positive regulation of chemokine production and B cell proliferation regulation, have been demonstrated to be highly correlated with the pathogenesis of COPD [63,64]. Furthermore, the significantly enriched pathways, e.g., MAPK [65], Wnt [66], and TGF-β [67] signaling, have been clarified as pathogenesis-related signaling during the progression of COPD.

The study has several limitations. Firstly, despite comprehensive dataset searching of GEO and ArrayExpress databases, insufficient publicly available cohorts containing miRNA expression profiles from the peripheral blood samples of COPD patients and healthy controls were included in the present study. The small sample size may lead to reduced stability and reliability of the diagnostic model. In addition, some essential clinical information was unavailable in GSE61741 and GSE70080 cohorts, including age, gender, and cigarette smoking status. Numerous studies have shown that smoking exposure is strongly associated with an increased risk of COPD [68,69]. Aging characterized by telomeres shortening and cellular senescence can contribute to COPD development [70]. Taking into account that various pathological factors may also affect transcriptional expression profiles, we need to consider other clinical information related to COPD pathogenesis besides smoking and aging, such as occupation, residential air pollution, and family history. Given that these clinical features can be accessed through the questionnaire, their incorporation may somewhat enhance the diagnostic performance of the model. Lastly, the diagnostic value of the miRNA pair-based model should be further validated in a population-based external cohort before translational clinical application.

To our knowledge, this is the first study to explore the potential value of circulating miRNA pairs for COPD diagnosis rather than the expression of single miRNAs or miRNA signature. We finally established the LightGBM-based machine learning model to provide novel strategies for early screening of the potential COPD population.

## Data Availability

All the analyzed data were obtained from the GEO database (https://www.ncbi.nlm.nih.gov/geo/, accessed on 1 February 2023). Processed data and codes used in this study are available from the corresponding authors upon reasonable request.

## Supplementary Materials

The following supporting information can be downloaded at: https://www.mdpi.com/article/10.3390/diagnostics13081440/s1, Figure S1: An illustrative example of the web application in which users predict their personalized diagnosis using the LightGBM model; Table S1: Differentially expressed miRNAs (|logFC| > 1.5, *p*-value < 0.05) in COPD samples compared with normal controls in GSE61741 and GSE70080 datasets; Table S2: Differentially expressed miRNAs (*p*-value < 0.05) in COPD samples compared with normal controls in GSE61741 and GSE70080 datasets; Table S3: Diagnostic miRNA pairs selected by ROC, information gain, MRMR, and Boruta algorithms; Table S4: The performance of the miRNA pair-based models developed with seven machine learning algorithms; Table S5: Information of five miRNA pairs and diagnosis in the FAHNMU cohort; Table S6: Significantly enriched terms of the five miRNA pairs.
