# Supplementary material for: A Diagnostic Classifier Based on Circulating miRNA Pairs for COPD Using a Machine Learning Approach
Source: Diagnostics (Basel). 2023 Apr 17;13(8):1440. doi: 10.3390/diagnostics13081440 (PMC10137826; doi:10.3390/diagnostics13081440)
Supplement: Supplementary file 1 [file diagnostics-13-01440-s001.zip › Supplementary figures.pdf]

A Diagnostic Classifier based on Circulating miRNA Pairs for COPD using a Machine Learning Approach

Shurui Xuan, et al.

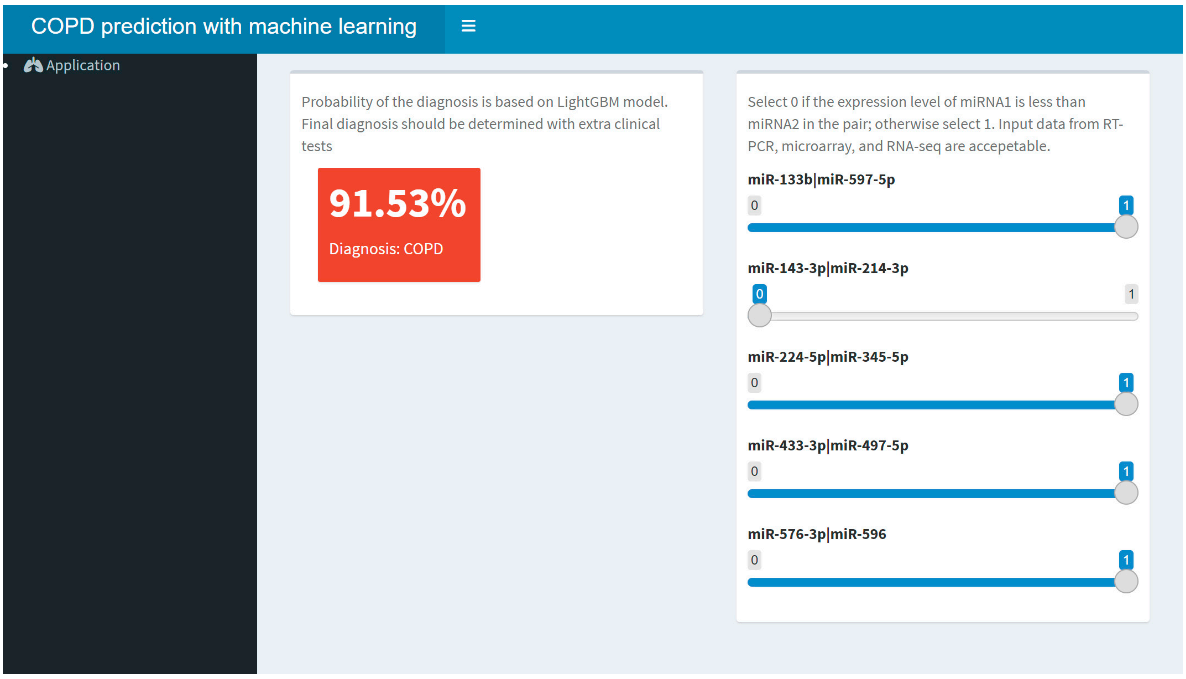

Figure S1. An illustrative example of the web application in which users predict their personalized diagnosis using the LightGBM model. Values of the five miRNA pairs can be interactively adjusted on the right panel. The predicted diagnosis and probability are shown in the box on the left panel
